# Role of brassinosteroids in rice spikelet differentiation and degeneration under soil-drying during panicle development

**DOI:** 10.1186/s12870-019-2025-2

**Published:** 2019-09-18

**Authors:** Weiyang Zhang, Jiayan Sheng, Yunji Xu, Fei Xiong, Yunfei Wu, Weilu Wang, Zhiqin Wang, Jianchang Yang, Jianhua Zhang

**Affiliations:** 1 Jiangsu Key Laboratory of Crop Genetics and Physiology/Jiangsu Key Laboratory of Crop Cultivation and Physiology, Agricultural College of Yangzhou University, Yangzhou, 225009 China; 2 Jiangsu Co-Innovation Center for Modern Production Technology of Grain Crops, Yangzhou University, Yangzhou, 225009 China; 3 Joint International Research Laboratory of Agriculture and Agri-Product Safety, the Ministry of Education of China, Yangzhou University, Yangzhou, 225009 Jiangsu China; 4 College of Bioscience and Biotechnology, Yangzhou University, Yangzhou, 225009 Jiangsu China; 5 Department of Biology, Hong Kong Baptist University, Hong Kong, China

**Keywords:** Ascorbic acid (AsA), Assimilate distribution, Brassinosteroids (BRs), Rice (*Oryza sativa* L.), Soil-drying, Spikelet differentiation and degeneration

## Abstract

**Background:**

Brassinosteroids (BRs) are a new group of plant hormones and play important roles in plant growth and development. However, little information is available if BRs could regulate spikelet development in rice (*Oryza sativa* L.) especially under soil-drying conditions. This study investigated whether and how BRs mediate the effect of soil-drying on spikelet differentiation and degeneration in rice. A rice cultivar was field-grown and exposed to three soil moisture treatments during panicle development, that is, well-watered (WW), moderate soil-drying (MD) and severe soil-drying (SD).

**Results:**

Compared with the WW treatment, the MD treatment enhanced BRs biosynthesis in young panicles, increased spikelet differentiation and reduced spikelet degeneration. The SD treatment had the opposite effects. Changes in expression levels of key rice inflorescence development genes (*OsAPO2* and *OsTAW1*), ascorbic acid (AsA) content, and activities of enzymes involved AsA synthesis and recycle, and amount of nonstructural carbohydrates (NSC) in young panicles were consistent with those in BRs levels, whereas hydrogen peroxide (H_2_O_2_) content showed opposite trend. Knockdown of the BRs synthesis gene *OsD11* or application of a BRs biosynthesis inhibitor to young panicles markedly decreased *OsAPO2* and *OsTAW1* expression levels, BRs and AsA contents, activities of enzymes involved AsA synthesis and recycle, NSC amount in rice panicles and spikelet differentiation but increased the H_2_O_2_ content and spikelet degeneration compared to the control (the wide type or application of water). The opposite effects were observed when exogenous BRs were applied.

**Conclusions:**

The results suggest that BRs mediate the effect of soil-drying on spikelet differentiation and degeneration, and elevated BRs levels in rice panicles promote spikelet development under MD by enhancing inflorescence meristem activity, AsA recycle and NSC partitioning to the growing panicles.

## Background

The panicle size, an important agronomic trait that makes valuable contributions to the grain productivity in rice (*Oryza sativa* L.), mainly depends on the number of spikelets on a panicle [1–4]. The final panicle size depends on the developmental processes of both spikelet differentiation and spikelet degeneration [3]. The phenomenon of young spikelet degeneration in cereal crops such as rice occurs frequently and is a serious physiological defect that causes grain yield loss [3, 5, 6]. Therefore, reduction or elimination of spikelet degeneration to increase spikelet number is a ‘scientific conundrum’ and is critical in increasing grain yield of cereals. Great effort has been made to increase spikelets differentiation and reduction or elimination of spikelet degeneration in rice by agronomic, genetic and molecular approaches [3–5, 7–10]. However, the mechanism involved in spikelet differentiation and degeneration remains unclear.

Drought is the most common abiotic stress inhibiting growth and development during the entire rice life cycle. Reproductive development is extremely vulnerable to water stress, leading to spikelet degeneration and yield loss [10–12]. In contrast, Yang et al. observed that moderate soil-drying during grain filling, which allows plants to rehydrate overnight and does not obviously inhibit photosynthesis, increases the grain-filling rate and grain weight of both rice and wheat (*Triticum aestivum* L) [13–15]. However, little is known whether and how moderate soil-drying during the panicle development could increase differentiation and decrease degeneration of rice spikelet.

Brassinosteroids (BRs) are a relatively recently discovered group of naturally occurring plant steroid hormones that are essential for normal plant growth, development and stress tolerance through producing an array of metabolism changes, and they have wide ranging biological activity and are widely distributed throughout whole plant [3, 16, 17]. However, it is little known whether BRs in young rice panicles respond to soil-drying during panicle development and thereby regulate spikelet differentiation and degeneration.

Usually, drought results in potentially damaging effects through the deleterious production of reactive oxygen species (ROS), such as hydrogen peroxide (H_2_O_2_), which is believed to be the main factor in damaging spikelet development of rice [3, 18–20]. However, moderate soil-drying during grain filling has been shown to reduce the H_2_O_2_ content in the grain, leading to increases in the grain-filling rate and grain weight in rice [21]. Our previous work showed that BRs in rice plants can mediate the effects of nitrogen fertilization on spikelet development by decreasing H_2_O_2_ levels during panicle development [3]. To eliminate ROS, plants have developed a complex AOS, including, enzymes and antioxidant molecules, such as ascorbic acid (AsA) [22, 23]. Besides, sugars are not only as a major source of carbon and energy but also have a signaling role in many physiological processes, including the network mechanism related to spikelet development in rice, and some key genetic and molecular switches recently identified from rice, that play critical role in regulating inflorescence development and grain yield [7, 10, 24]. It is interesting to know whether BRs can regulate spikelet development by manipulating AsA and sugars metabolism, and key inflorescence development genes expressions in young rice panicles under soil-drying during panicle development.

The objective of this study was to test the hypothesis that BRs mediate the effect of soil-drying on rice spikelet differentiation and degeneration. Three soil-drying treatments, well-watered (WW), moderate soil-drying (MD) and severe soil-drying (SD), were conducted during panicle development. The temporal patterns of BRs levels/ or BRs synthesis gene expression, and the expressions of key genes involved in inflorescence development, changes in the levels of the AsA and its synthesis and metabolism related genes expressions/ or enzymes activities, and the H_2_O_2_ content in young panicles were determined. Transgenic plants and chemical regulators were used to verify the role of BRs in rice spikelet differentiation and degeneration.

## Results

### Leaf and panicle osmolality, leaf photosynthetic rate and leaf area index (LAI)

**Fig. 1:**
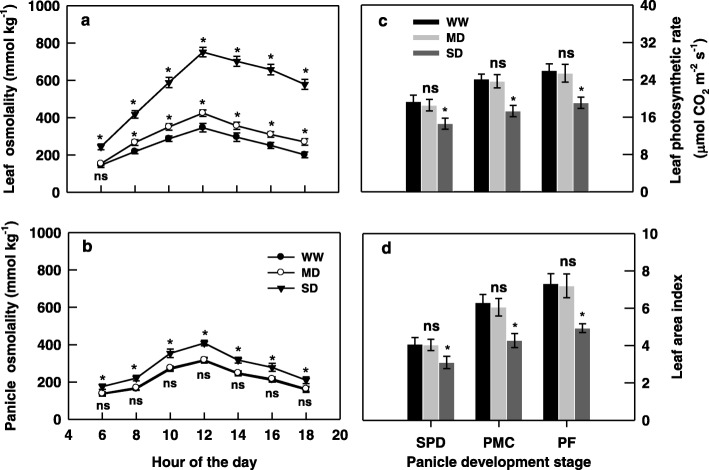
Changes in osmolality of upmost fully-expanded leaves (**a**) and panicles (**b**), photosynthetic rate of the upmost fully-expanded leaves (**c**) and leaf area index (LAI) (**d**) of the rice cultivar YD-6 under well-watered (WW), moderate soil-drying (MD) and severe soil-drying (SD) treatments. SPD, PMC, and PF represent spikelet primordium differentiation, pollen mother cells meiosis, and pollen filling, respectively. Vertical bars represent standard error of the mean (since the panicle and leaf osmolality at the three stages showed the same tendencies, the data are presented on average of the three stages and *n* = 18; *n* = 6 for leaf photosynthetic and LAI) where these exceed the size of the symbol. The asterisk (*) represents significant difference (*P* = 0.05) between the MD regime and the WW regime or between the SD regime and the WW regime within the same measurement date. “ns” represents non-significant at *P* = 0.05 between the MD regime and the WW regime

Leaf osmolality showed a small change during the day for WW plants and was significantly increased during the day for MD and SD plants, with a larger increase for the SD plants than for the WW plants (Fig. 1a). The predawn (0600 h) leaf osmolality of MD plants were not significantly different from that of WW plants but was significantly lower than that of SD plants (Fig. 1a). In contrast to the large increase in leaf osmolality, the diurnal changes in panicle osmolality remained constant at midday (Fig. 1b). Panicle osmolality was not significantly different between WW and MD plants, whereas it was significantly higher in SD plants (Fig. 1b), indicating that MD plants were able to rehydrate overnight and well maintain the water status in their young panicles during the day, whereas SD plants were seriously inhibited by soil-drying.

Consistent with plant water status, the photosynthetic rate of leaves and LAI did not differ significantly between WW and MD plants, whereas they were significantly lower in SD plants (Fig. 1c, d).

### Nonstructural carbohydrates (NSC) accumulation and distribution

**Fig. 2:**
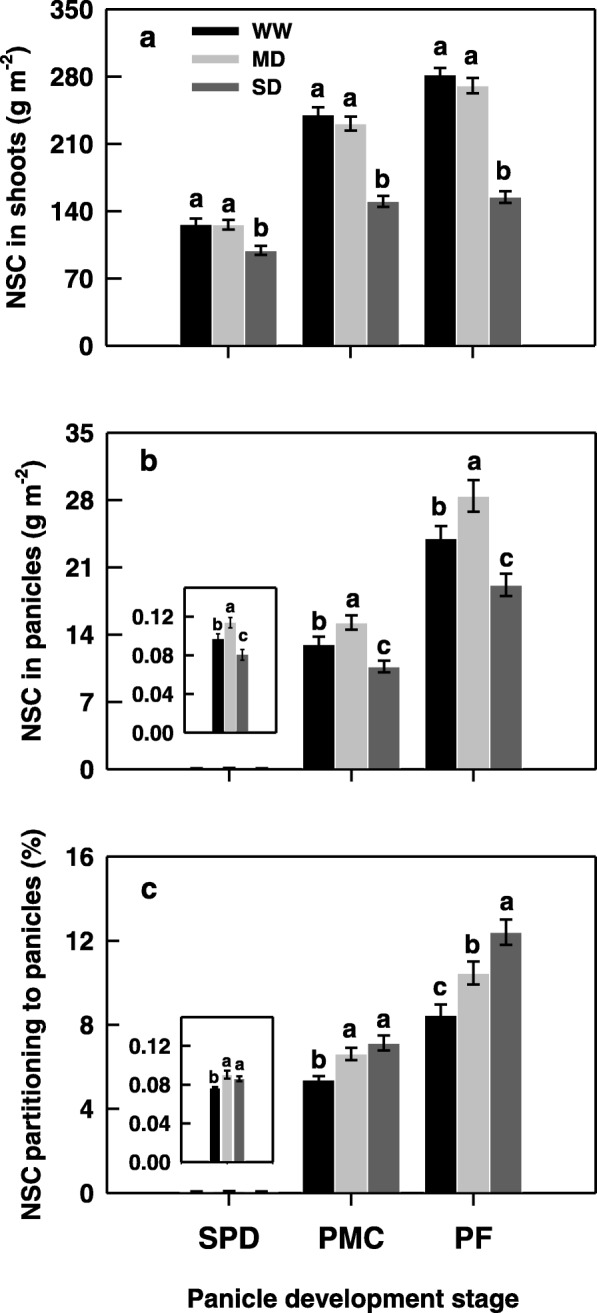
Changes in non-structural carbohydrate (NSC) distribution of the rice cultivar YD-6 (**a**-**c**) under well-watered (WW), moderate soil-drying (MD) and severe soil-drying (SD) treatments. SPD, PMC, and PF represent spikelet primordium differentiation, pollen mother cells meiosis, and pollen filling, respectively. Vertical bars represent ± standard error of the mean (*n* = 6) where these exceed the size of the symbol. Different letters above the bars indicate the least significant difference at *P* = 0.05 within the same measurement date

Change in the amount of NSC in the shoot was consistent with leaf photosynthetic rate under various soil moisture treatments. The amount of NSC in the shoot exhibited no significant difference between the WW and MD treatments, whereas was greatly decreased under the SD treatment (Fig. 2a). The MD treatment markedly increased the amount of NSC in young panicles, and the SD treatment significantly decreased it when compared with the WW treatment (Fig. 2b). Opposite to the NSC accumulation in the shoot, the rate of NSC partitioning to the growing young panicle was increased with the increase in soil-drying degrees (Fig. 2c). The NSC was most partitioned to the growing young panicle under the SD, intermediate under the MD, and the least in the WW treatment, suggesting that soil-drying during panicle development enhances the partitioning of assimilates from vegetative tissues to growing young panicles of rice.

### Changes in genes expressions, enzymes activities and contents of BRs, AsA and H_2_O_2_

**Fig. 3:**
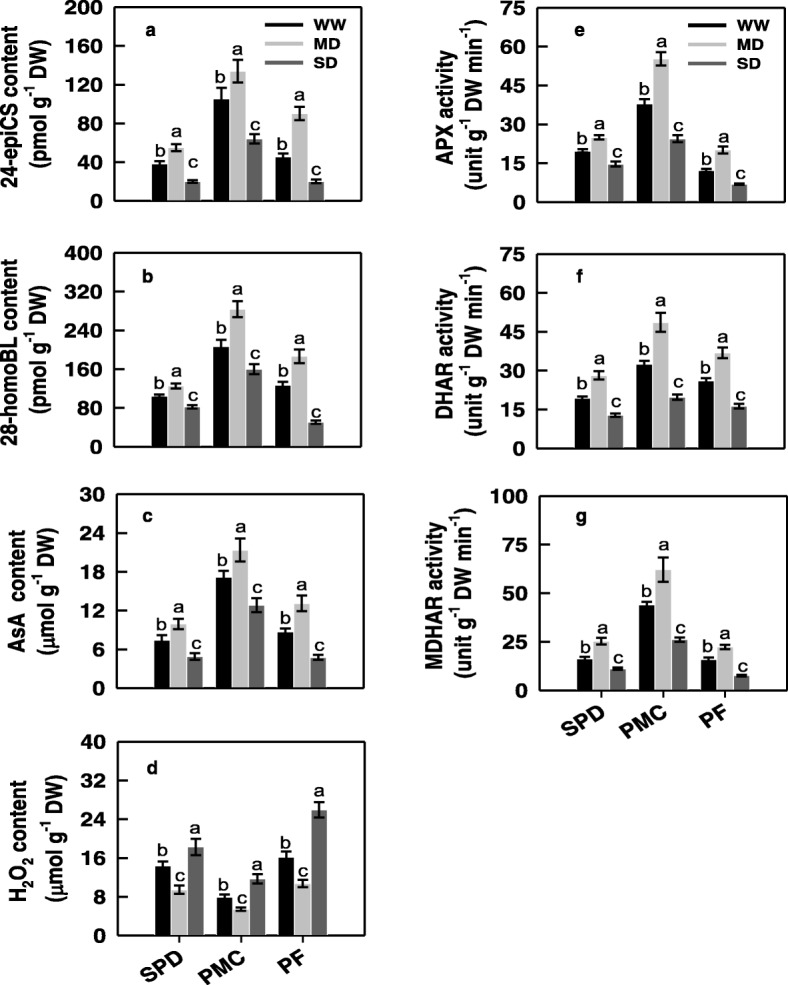
Changes in contents of brassinosteroids (BRs) (**a**, **b**), ascorbic acid (AsA) (**c**) and hydrogen peroxide (H_2_O_2_) (**d**), and activities of ascorbic acid peroxidase (APX) (**e**), dehydroascorbate reductase (DHAR) (**f**) and monodehydroascorbate reductase (MDHAR) (**g**) in young panicle of the rice cultivar YD-6 under well-watered (WW), moderate soil-drying (MD) and severe soil-drying (SD) treatments. SPD, PMC, and PF represent spikelet primordium differentiation, pollen mother cells meiosis, and pollen filling, respectively. Vertical bars represent ± standard error of the mean (*n* = 6) where these exceed the size of the symbol. Different letters above the bars indicate the least significant difference at *P* = 0.05 within the same measurement date

The MD treatment significantly increased, whereas the SD treatment significantly decreased, the expression levels of the key BRs synthesis gene *OsD11,* key inflorescence development genes *OsAPO2* and *OsTAW1*, AsA synthesis and metabolic related genes *OsMPG1*, *OsDHAR1*, *OsMDHAR3*, *OsAPX1*, *OsAPX2*, and key sugar partitioning and metabolism gene *OsCSA* in rice young panicles (Additional file 1: Fig. S1a-c and Fig. S3a). Changes in the activities of ascorbic acid peroxidase (APX), dehydroascorbate reductase (DHAR) and monodehydroascorbate reductase (MDHAR), contents of 24-epiCS, 28-homoBL and AsA in young panicles were very similar to those of *OsD11*, *OsMPG1*, *OsDHAR1*, *OsMDHAR3*, *OsAPX1*, *OsAPX2*, *OsAPO2*, *OsTAW1***,** and *OsCSA* expression levels. They were significantly higher in the MD treatment and significantly lower in the SD treatment than in the WW treatment (Fig. 3a-c, e-g; Additional file 1: Fig. S1a-c and Fig. S3a). In contrast to the AsA content, the H_2_O_2_ content was significantly decreased under the MD, and significantly increased under the SD treatment (Fig. 3d).

### Spikelet development and grain yield

**Fig. 4:**
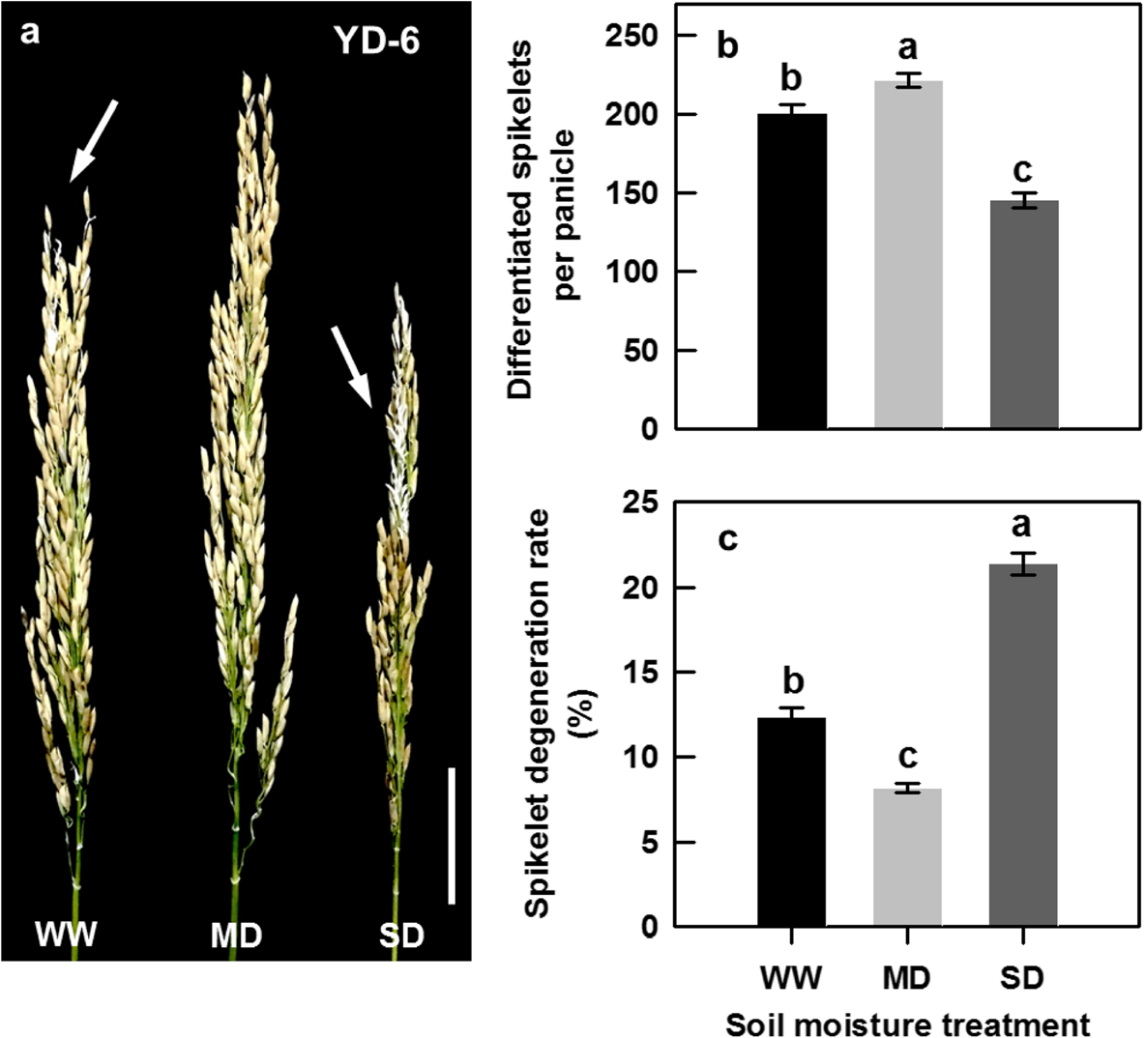
Spikelet development of the rice cultivar YD-6 under well-watered (WW), moderate soil-drying (MD) and severe soil-drying (SD) treatments. (**a**) Panicle morphology of YD-6 under various soil-drying treatments at maturity. Bar = 5 cm; (**b**, **c**) Comparisons of differentiated spikelets per panicle and spikelet degeneration rate under various soil-drying treatments. The arrow indicates the obvious degenerated spikelets in a. Vertical bars represent ± standard error of the mean (*n* = 6) where these exceed the size of the symbol. Different letters above the bars indicate the least significant difference at *P* = 0.05

Compared with the WW treatment, the MD treatment significantly increased differentiated spikelet number and panicle size, and significantly decreased spikelet degeneration rate. The SD treatment showed the opposite effects (Fig. 4a-c). As a result, the MD treatment significantly increased total number of spikelets and grain yield, whereas the SD markedly decreased those relative to the WW treatment (Table 1).

**Table 1:** Grain yield and yield components of the rice cultivar YD-6 subjected to various soil moisture treatments ^1)^

Items	Soil moisture treatment		
	WW	MD	SD
Total number of spikelets (× 10^3^ m^−2^)	29.5 ± 0.69 b ^2)^	33.6 ± 0.93 a	18.9 ± 0.38 c
Fully filled grains (%)	88.8 ± 2.15 a	89.9 ± 1.12 a	72.7 ± 3.04 b
1000-grain weight (g)	27.8 ± 0.53 a	27.5 ± 0.54 a	28.0 ± 0.68 a
Grain yield (g m^− 2^)	730 ± 32.3 b	828 ± 22.3 a	385 ± 25.6 c

^1)^The treatments were well-watered (WW), moderate soil-drying (MD) and severe soil-drying (SD) during young panicle development

^2)^Data are means ± standard error of six independent measurements and values with different letters indicate statistical difference at *P* = 0.05 within the same row

### Effects of MD on RNA interference (RNAi) line

**Fig. 5:**
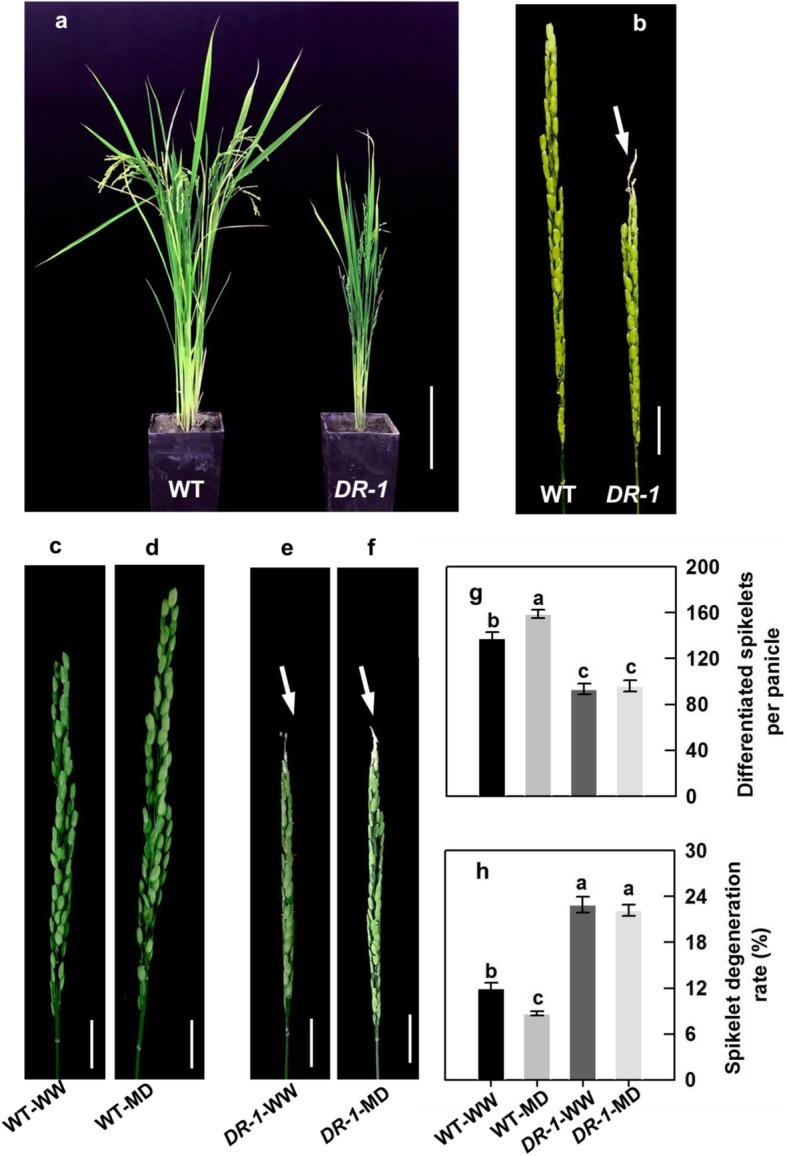
Phenotypes of wild-type (WT) and *D11-RNAi* (*DR-1*) plants under well-watered (WW) and moderate soil-drying (MD) treatments. (**a**) Gross morphology of an adult WT (Zhonghua 10, ZH10) and *DR-1* plants. Bar = 30 cm; (**b**) Comparison of representative panicle of *DR-1* with the WT at pollen filling stage. (**c-f**) Panicle morphology of WT and *DR-1* under various soil-drying treatments during panicle development. Bars = 2 cm; (**g**, **h**) Comparisons of differentiated spikelets number and spikelet degeneration rate of *DR-1* with the WT under various soil soil-drying treatments. The arrow indicates the obvious degenerated spikelets in b, e and f. Vertical bars represent ± standard error of the mean (*n* = 6) where these exceed the size of the symbol. Different letters above the bars indicate the least significant difference at *P* = 0.05

**Fig. 6:**
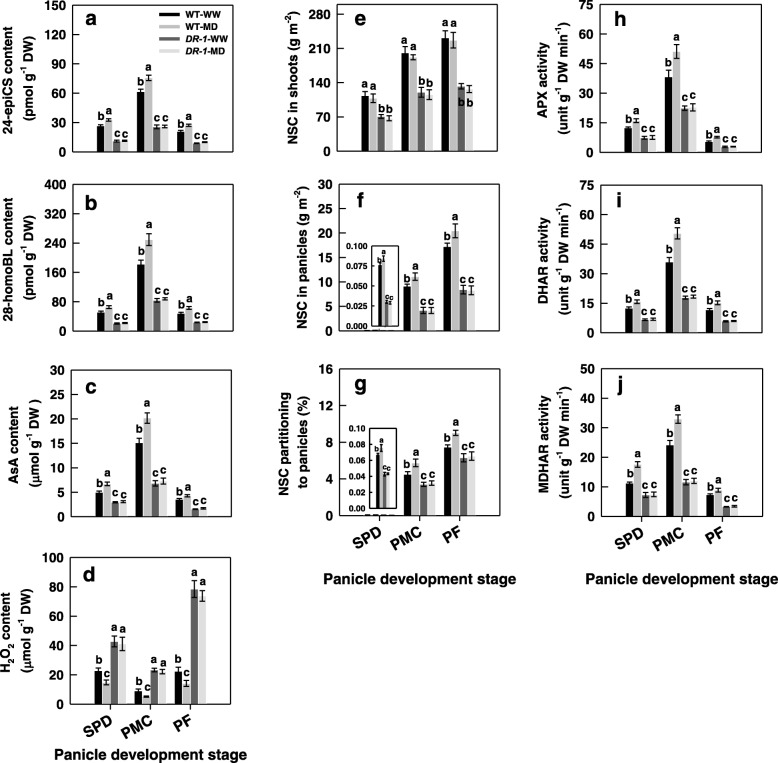
Changes in contents of brassinosteroids (BRs) (**a**, **b**), ascorbic acid (AsA) (**c**) and hydrogen peroxide (H_2_O_2_) (**d**), non-structural carbohydrate (NSC) distribution (**e-g**), and activities of ascorbic acid peroxidase (APX), dehydroascorbate reductase (DHAR) and monodehydroascorbate reductase (MDHAR) (**h-j**) in young rice panicles of WT and *DR-1* under well-watered (WW) and moderate soil-drying (MD) treatments. SPD, PMC, and PF represent spikelet primordium differentiation, pollen mother cells meiosis, and pollen filling, respectively. Vertical bars represent ± standard error of the mean (*n* = 6) where these exceed the size of the symbol. Different letters above the bars indicate the least significant difference at *P* = 0.05 within the same measurement date

To understand the molecular mechanism through which BRs regulate spikelet development in rice, we used an RNAi line of transgenic rice in which the *OsD11* gene encoding a cytochrome P450 enzyme (CYP724B1) involved in BRs biosynthesis had reduced expression (*D11-RNAi 1*, called *DR-1*), and its wild type (WT) was the inbred *japonica* rice cultivar Zhonghua 10 (ZH10). The *DR-1* plants exhibited a typical phenotype of rice plants with reduced BRs levels, that is, the plant size and degree of lamina joint bending were both lower than those in the wide type (WT) plants (Fig. 5a). The *DR-1* plants displayed a significant reduction in the differentiated spikelet number and significant increases in spikelet degeneration rate compared to the WT plants (Fig. 5b-h). Consistent with the phenotype of the *DR-1* plants, the *OsD11*, *OsAPO2*, *OsTAW1*, *OsCSA*, *OsMPG1*, *OsDHAR1*, *OsMDHAR3*, *OsAPX1* and *OsAPX2* expression levels, BRs (24-epiCS and 28-homoBL) and AsA contents, and NSC amount, and APX, DHAR and MDHAR activities were significantly lower, whereas the H_2_O_2_ content was significantly higher, in *DR-1* panicles than in WT panicles in the WW and MD treatments (Fig. 6a-j; Additional file 1: Fig. S2a-f and Fig. S3b).

The MD treatment significantly increased expression levels of *OsD11*, *OsAPO2*, *OsTAW1*, *OsCSA*, *OsMPG1*, *OsDHAR1*, *OsMDHAR3*, *OsAPX1* and *OsAPX2*, contents of BRs (24-epiCS and 28-homoBL), AsA and NSC, activities of APX, DHAR and MDHAR, and differentiated spikelet number, whereas significantly decreased H_2_O_2_ content in young panicles and spikelet degeneration rate of WT plants. However, the MD treatment had no significant effects on these characteristics in the *DR-1* plants (Fig. 5a-h and Fig. 6a-j; Additional file 1: Fig. S2a-f and Fig. S3b).

### Effect of chemical application

**Fig. 7:**
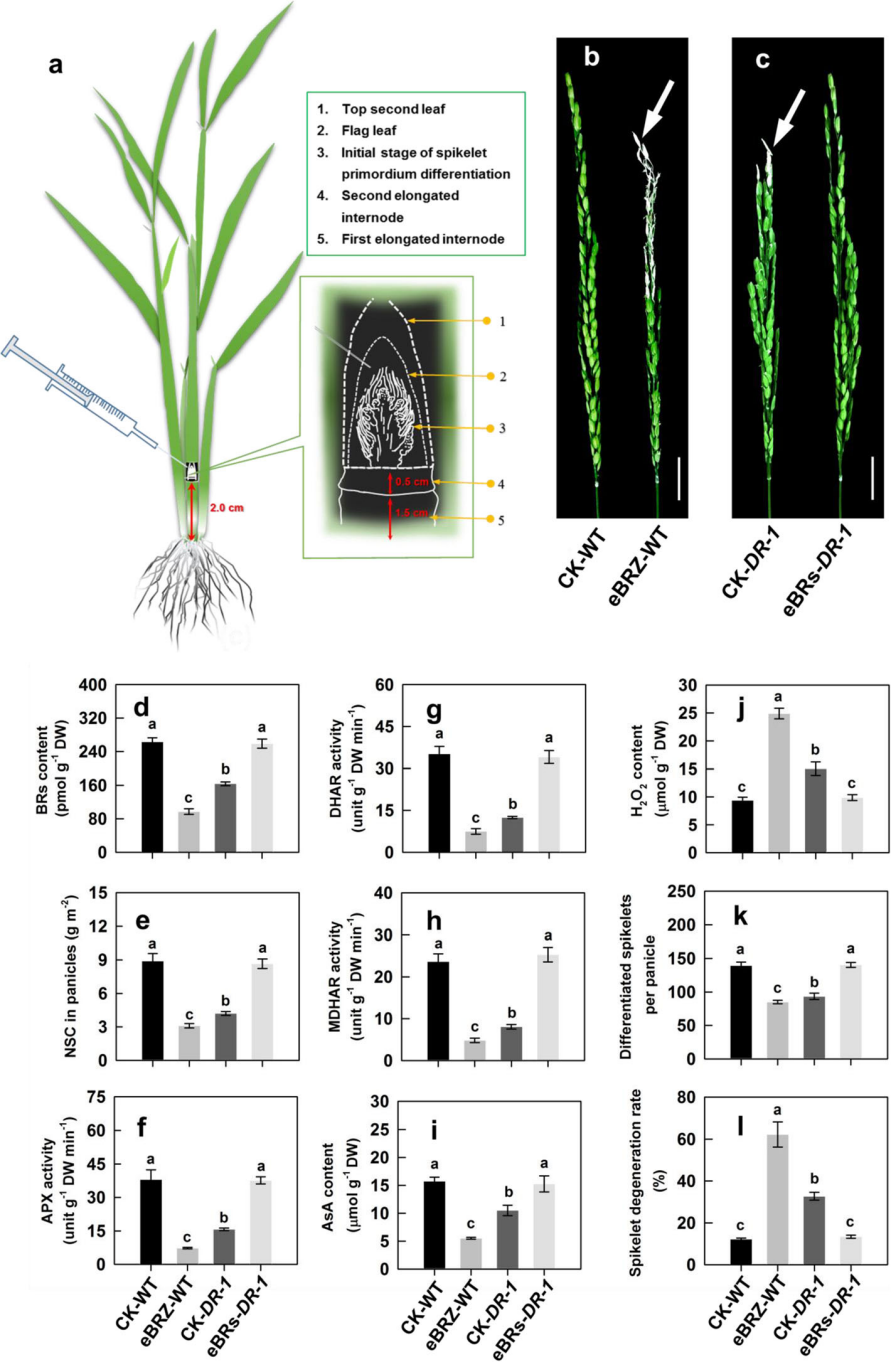
Effect of exogenous brassinosteroids (BRs) and brassinazole (BRZ) on physiological traits and spikelet development of WT and *DR-1* plants. (**a**) Schematic diagram of the initial stage of spikelet primordium differentiation and the injection site in the chemical treatment experiment; (**b**, **c**) Panicle phenotypes of WT and *DR-1* under various chemical regulators treatments. Bars = 2 cm. The arrow indicates the obvious degenerated spikelets; (**d**-**j**) Changes in contents of BRs (24-epiCS + 28-homoBL), NSC, AsA and H_2_O_2_, activities of APX, DHAR, and MDHAR in young rice panicles; (**k, l**) Spikelet development under various chemical regulators treatments. CK: Panicles received deionized water; eBRZ: Panicles received 10 nmol L^− 1^ brassinazole (BRZ, an inhibitor of BRs biosynthesis); eBRs: Panicles received 10 nmol L^− 1^ BRs (24-epiCS + 28-homoBL). Vertical bars represent ± standard error of the mean (*n* = 6) where these exceed the size of the symbol. Different letters above the bars indicate the least significant difference at *P* = 0.05

Compared with no chemical application (CK), the application of brassinazole (BRZ) (an inhibitor of BRs biosynthesis) to WT or YD-6 panicles in the WW treatment significantly decreased the expression levels of *OsAPO2*, *OsTAW1*, *OsCSA*, *OsMPG1*, *OsDHAR1*, *OsMDHAR3*, *OsAPX1* and *OsAPX2*, contents of BRs (24-epiCS + 28-homoBL), AsA and NSC, and activities of APX, DHAR and MDHAR, significantly increased H_2_O_2_ content in young panicles, and significantly decreased the differentiated spikelet number and increased spikelet degeneration rate. The opposite effects were observed when BRs (24-epiCS + 28-homoBL) were applied to the panicles of *DR-1* or SD treated-YD-6 (Fig. 7a-i; Additional file 1: Fig. S4a, b; Fig. S5a-d and Fig. S6a-i). In addition, applied H_2_O_2_ to WT or YD-6 panicles in the WW treatment could significantly increase endogenous H_2_O_2_ content in the panicles, and decrease the differentiated spikelet number and increase spikelet degeneration rate, whereas application of AsA or AsA combined with sucrose to *DR-1* or SD treated-YD-6 panicles could significantly decrease endogenous H_2_O_2_ content in the panicles, and consequently, increase the differentiated spikelet number and decrease spikelet degeneration rate (Additional file 1: Fig. S7a-f).

## Discussion

### Effect of soil-drying during panicle development on rice spikelet differentiation and degeneration

The growth period from meiosis to flowering in cereal crops is generally thought to be very sensitive to environmental changes, and spikelet degeneration is increasingly subjected to drought during this period [10–12]. In our study, a SD treatment imposed during panicle development significantly decreased spikelet differentiation and increased spikelet degeneration (Fig. 4a-c). However, the MD treatment not only promoted spikelet differentiation, but also effectively suppressed spikelet degeneration, leading to more spikelets per rice panicle, a higher grain yield than the WW treatment (Table 1; Fig. 4a-c). We argue that the susceptibility of rice to soil-drying during panicle development varies with the degree of soil drying, and that moderate soil-drying, that is, soil water potential is not lower than − 15 kPa or the osmolality of a young panicle is no more than 300 mmol kg^− 1^, benefits spikelet growth and development. This finding would have great significance to improve spikelet development of rice, especially for the newly bred ‘super’ rice which exhibits more numerous spikelets potential and higher frequency of degenerated spikelets [3, 5, 10].

### BRs mediate the effect of soil-drying on rice spikelet differentiation and degeneration

The mechanism by which soil-drying, especially MD improves panicle development in rice remains unclear. It is proposed that the increase in BRs biosynthesis would enhance the role of BRs in regulating plant growth and development and responsing to environmental stresses [3, 16, 17, 25–28]. The gene *OsD11* is believed to belong to the cytochrome P450 superfamily and function in the BRs biosynthesis pathway in rice, and over expression of *OsD11* at a suitable level could increase grain size, grain number and grain yield in rice [29, 30]. The MD significantly increased, whereas the SD markedly decreased, the *OsD11* expression level and contents of 24-epiCS and 28-homoBL in young rice panicles (Fig. 3a, b; Additional file 1: Fig. S1a-c). The *OsD11* expression level, 24-epiCS and 28-homoBL contents of young panicles were highly consistent with differentiated spikelet number and showed a trend opposite to that of the spikelet degeneration rate (Fig. 3a, b and Fig. 4a-c; Additional file 1: Fig. S1a-c). The *DR-1* plants showed significant reduction in *OsD11* expression level, 24-epiCS and 28-homoBL contents and differentiated spikelet number, and significant increases in spikelet degeneration rate compared with the WT plants in both the WW and MD treatments. The results herein also showed that the MD treatment significantly increased the *OsD11* expression level, BRs (24-epiCS and 28-homoBL) contents and differentiated spikelet number, and decreased the spikelet degeneration rate of the WT plants, whereas exhibited no distinct effect on the *OsD11* expression level, BRs content, spikelet differentiation and degeneration of *DR-1* plants (Fig. 5a-h and Fig. 6a, b; Additional file 1: Fig. S2a). When BRs (24-epiCS + 28-homoBL) were applied to young panicles of *DR-1* or SD treated-YD-6, the BRs levels and differentiated spikelet number were significantly increased, whereas the spikelet degeneration rate was significantly decreased when compared to the control. The opposite effects were observed when BRZ, an inhibitor of BRs biosynthesis, was applied to young panicles of WT or WW treated-YD-6 (Fig. 7a-d, k, l; Additional file 1: Fig. S6a, h, i). The results imply that the more enhanced BRs biosynthetic activity (the *OsD11* expression level) in rice panicles under MD contributes to higher levels of 24-epiCS and 28-homoBL, leading to more differentiated spikelet number, and decreases in spikelet degeneration rate, and consequently, to a significant increase in grain yield. In contrast, the decreases in BRs synthesis in the SD treatment inhibited spikelet development and, as a result, led to a low grain yield.

### Mechanism underlying BRs regulate rice differentiation and degeneration

Little is known about how BRs regulate spikelet development in rice. Previous studies have shown that the spikelet number per panicle is closely correlated with amount of the available NSC in rice [24, 31]. The results herein showed that soil-drying promoted the re-allocation of NSC from the vegetative tissues to growing panicles (Fig. 2a-c and Fig. 6e-g). The SD markedly increased spikelet degeneration rate, decreased the amount of NSC in young panicles, differentiated spikelets and grain yield although the rate of NSC partitioning to the growing young panicle was increased, implying that the growing panicle from an enhanced NSC remobilization could not compensate for the photosynthesis loss during the panicle development under such a treatment (Fig. 1c, d; Fig. 2a-c and Fig. 4a-c). Over-expression of BRs biosynthetic or BRs-signaling genes can increase expression of *OsCSA* which encodes a MYB domain protein and directly triggers expression of genes relating to sugar partitioning, cleavage and uptake and starch synthesis in reproductive organs, and increased seed size and weight in rice [32, 33]. Moreover, some genes such as *OsAPO2* and *OsTAW1* are known to be critical for controlling rice spikelet numbers by inhibiting the precocious transition from inflorescence meristem to spikelet meristem, and their overexpression displayed a prolonged inflorescence meristem activity and delayed spikelet specification, causing prolonged branch development and increased spikelet numbers [7]. We found, however, the MD not only substantially enhanced the rate of NSC partitioning to the growing panicles, but also improved the BRs (24-epiCS and 28-homoBL) contents, expression levels of *OsAPO2* and *OsTAW1* and differentiated spikelet number, and decreased spikelet degeneration rate of both YD-6 and WT, whereas the panicles of *DR-1* or SD treated-YD-6, which showed reduction in *OsD11* expression, BRs contents, expression levels of *OsAPO2*, *OsTAW1* and *OsCSA*, and NSC amount, resulted in less differentiated spikelet number and more spikelet degeneration rate compared with those in control conditions, however, the MD had no distinct effect on those of *DR-1* plants (Fig. 2a-c; Fig. 3a, b; Fig. 4a-c; Fig. 5a-h and Fig. 6a, b, e-g; Additional file 1: Fig. S1a-c; Fig. S2a, b and Fig. S3a, b). When BRs (24-epiCS + 28-homoBL) were applied to the young panicles of *DR-1* or SD treated-YD-6, the expression levels of *OsAPO2*, *OsTAW1* and *OsCSA*, and NSC amount in young panicles were significantly increased, the spikelet degeneration rate was decreased and differentiated spikelet number was increased, whereas application of an inhibitor of BRs biosynthesis to the young panicles of WT or WW treated-YD-6 showed the opposite effects (Fig. 7a-e, k, l; Additional file 1: Fig. S4a, b; Fig. S5a-d and Fig. S6a, b, h, i). Therefore, we conclude that if a soil-drying is applied during panicle development to a degree that overnight rehydration can be completed, water retention in growing panicle and photosynthesis is not too severely inhibited, then the growing panicle can get more NSC from vegetative tissues and can outweigh the small loss of photosynthesis, and also get prolonged inflorescence meristem activity by increasing BRs levels in growing rice panicles.

**Fig. 8:**
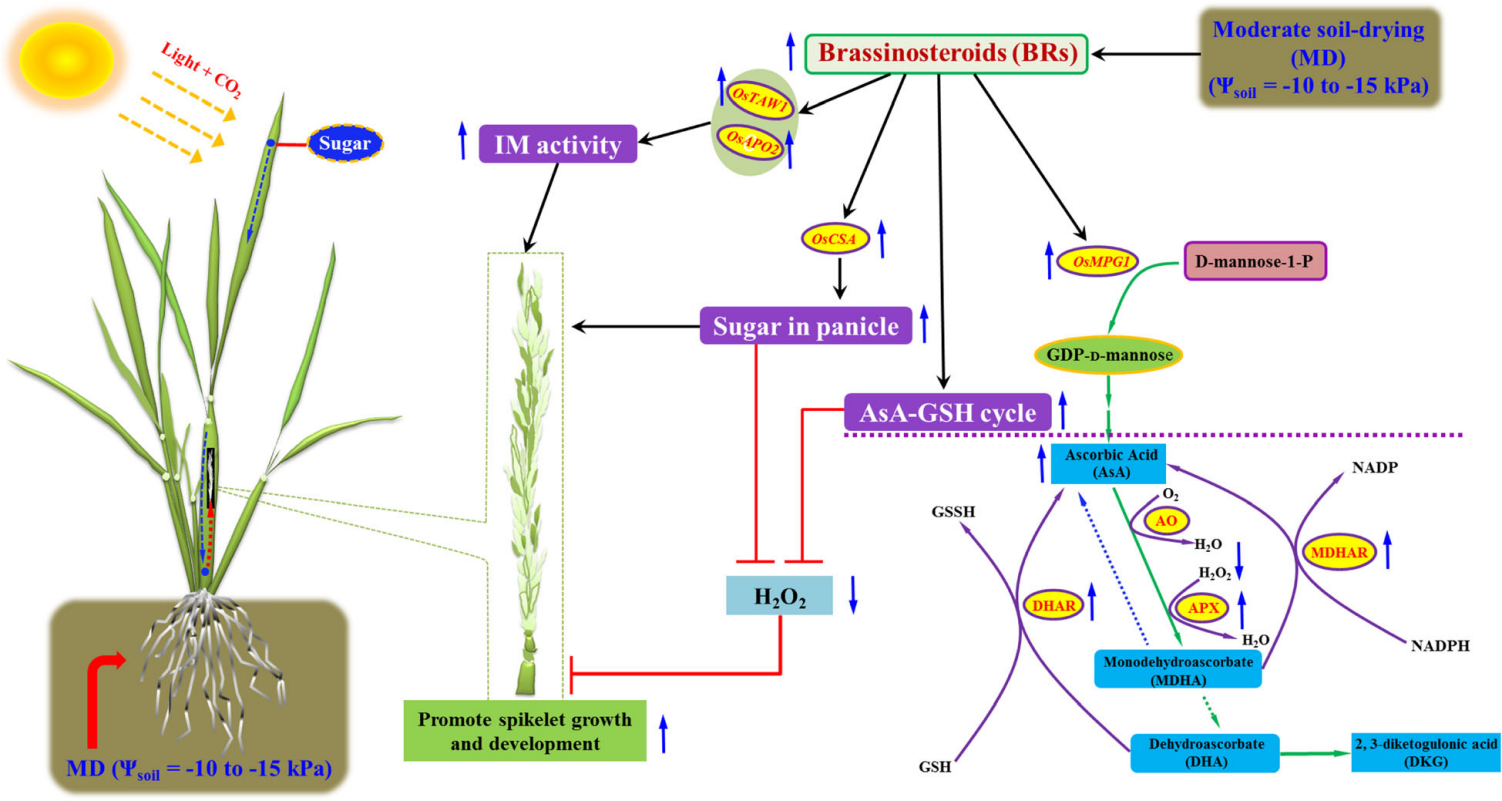
The descriptive model of brassinosteroids (BRs) function in spikelet development under moderate soil-drying (MD) treatment during rice panicle development. Under MD treatment, photosynthesis is not severely inhibited, and the growing panicle can get more NSC from vegetative tissues, and also get prolonged inflorescence meristem (IM) activity, and the AsA synthesis and recycle (AsA-GSH cycle) were enhanced to protect spikelet growth and development from the deleterious effect of H_2_O_2_ in panicles by increasing BRs level in growing panicles. The black arrow “→” indicates enhancement, while red “˧” indicates inhibition. Up-regulated items under the MD treatment are marked with upward blue arrows, and down-regulated items under the MD treatment are marked with downward blue arrows

AOS include antioxidant enzymes and antioxidant molecules such as AsA play a fundamental role in scavenging ROS due to its powerful antioxidant properties, and AsA can be modified by exogenous BRs treatment [3, 22, 28, 34]. Moreover, the metabolic responses to drought stress are an indirect response to oxidative stress, rather than direct responses to drought stress, and excessive production and accumulation of ROS such as H_2_O_2_ can damage the cellular membrane and cause rice spikelet damage [35]. In plants, GDP-D-mannose as a precursor of AsA, can be synthesized by GDP-D-mannose pyrophosphorylase (GMPase) [36]. It is proposed that AsA as a major antioxidant, is firstly oxidized into an unstable short-lived monodehydroascorbate (MDHA) by ascorbic acid oxidase (AO) and APX [37], and then some MDHAs are reduced to AsA by MDHAR, and other MDHAs are converted into AsA and dehydroascorbate (DHA) through spontaneous disproportionation, and DHA must be converted to AsA by DHAR in a reaction requiring reduced glutathione (GSH) [38]. In this process, DHAR is a key factor in maintaining a reduced pool of AsA [18]. We observed that the panicles of *DR-1* or SD treated-YD-6, which showed a significantly less differentiated spikelet number and higher spikelet degeneration rate, were accompanied by decreased BRs contents, expression levels of *OsMPG1*, *OsDHAR1*, *OsMDHAR3*, *OsAPX1* and *OsAPX2*, lower activities of APX, DHAR and MDHAR, and higher content of H_2_O_2_ than the panicles of WT or WW treated-YD-6, whereas those in the *DR-1* panicles were not significantly affected by the MD treatment (Fig. 3a-g; Fig. 4a-c; Fig. 5a-h and Fig. 6a-d, h-j; Additional file 1: Fig. S1a-c; Fig. S2c-f; Fig. S4a, b and Fig. S6c-i). Application of BRs (24-epiCS + 28-homoBL) to young panicles of *DR-1* or SD treated-YD-6 elevated the BRs content there. Simultaneously, the expression levels of *OsMPG1*, *OsDHAR1*, *OsMDHAR3*, *OsAPX1* and *OsAPX2*, the activities of APX, DHAR and MDHAR and AsA content in rice panicles were markedly increased, whereas the H_2_O_2_ content was considerably decreased, and as a result, the differentiated spikelet number was increased, and the spikelet degeneration rate was significantly decreased. The opposite effects were observed when BRZ was applied to the young panicles of WT or WW treated-YD-6 (Fig. 7a-d, f-l; Additional file 1: Fig. S4a, b and Fig. S6a, c-i). Furthermore, soluble sugars can also scavenge the deleterious effect induced by H_2_O_2_ [31]. We also observed that the effect of applying H_2_O_2_ on spikelet development was very similar with that of applying BRZ, and the effects of applying AsA or AsA combined with sucrose on H_2_O_2_ level and spikelet development were very similar with that of applying BRs, and applying AsA combined with sucrose showed the better effects to decrease H_2_O_2_ accumulation in young panicle and enhance spikelet development than only AsA was applied (Additional file 1: Fig. S7a-e). These results indicate that the elevated BRs levels under the MD treatment could enhance AsA synthesis and recycle, and NSC remobilization to protect spikelet growth and development from the deleterious effect of H_2_O_2_ (Fig. 8).

It is noteworthy that the responses of *indica* cultivar (YD-6) and *japonica* cultivar (ZH10) to the MD and BRs application were very similar. Furthermore, treatment with exogenous BRs, the injury of SD on rice spikelet development could be relieved effectively. These results demonstrate that an increase in BRs biosynthesis is an important approach to enhance spikelet development and grain yield, and MD imposed during the young panicle development period is an effective technology to increase spikelet differentiation and decrease spikelet degeneration in rice.

## Conclusion

Moderate soil-drying imposed during the young panicle development period can increase spikelet differentiation and decrease spikelet degeneration of rice. The enhanced spikelet development under MD is closely associated with an increase in BRs biosynthesis in young rice panicles. In contrast, the severe soil-drying decreases BRs synthesis, and consequently, results in the decrease in spikelet differentiation and increase in spikelet degeneration. BRs mediate the effect of soil-drying on spikelet differentiation and degeneration by regulating inflorescence meristem activity, AsA recycle and NSC partitioning to the growing panicles.

## Methods

### Materials and culture conditions

The experiment was conducted at a research farm of Yangzhou University, Jiangsu Province, China (32°30′N, 119°25′E), during the rice-growing season (May–October). The soil was a sandy loam (Typic Fluvaquent, Entisol, US classification) that contained 24.2 g kg^− 1^ organic matter, 101.5 mg kg^− 1^ alkali-hydrolysable nitrogen (N), 34.2 mg kg^− 1^ Olsen phosphorus and 68.1 mg kg^− 1^ exchangeable potassium. The field-capacity soil moisture content measured gravimetrically at a constant drainage rate was 0.187 g g^− 1^, and the bulk density of the soil was 1.34 g cm^− 3^. A rice (*Oryza sativa* L.) cultivar currently used in local production, namely, Yangdao-6 (YD-6, an inbred *indica* cultivar), was grown in the field. Seeds were sown in the paddy field on 11 May. Thirty-day-old seedlings were then transplanted into the field with a hill spacing of 0.25 m × 0.16 m and two seedlings per hill. Nitrogen (N, 80 kg ha^− 1^ as urea), phosphorus (30 kg ha^− 1^ as single superphosphate) and potassium (40 kg ha^− 1^ as KCl) were applied and incorporated before transplanting. N as urea was also applied at mid-tillering (40 kg ha^− 1^), panicle initiation (40 kg ha^− 1^), and at spikelet primordium differentiation (SPD) (40 kg ha^− 1^). The developmental stages were observed by frequent inspections of meristems and leaf remainder (LR) as described by Ling et al. (1983) [39]. Panicle initiation was defined as the first appearance of a differentiated apex (LR: 4.0–3.5) and SPD as the appearance of glumous flower primordia at the tips of elongating primary rachis branches (the length of young panicles was approximately 1.0–1.5 mm; LR: 2.0–1.6). Except for drainage at the end tillering (12–15 July), the water level in the field was keep at the 1–2 cm until the onset of panicle initiation (21–25 July) when soil-drying treatments were initiated. The cultivar headed on 24–26 August (50% of plants) and was harvested on 15–16 October.

### Treatments

The experiment followed a one by three (1 cultivar and 3 levels of soil moisture) factorial design with three treatments. Each of the treatments included three replicate plots in a complete randomized block design. The plot size was 5 m × 6 m, and the plots were separated by an alley 1-m wide with plastic film inserted into the soil to a depth of 50 cm to form a barrier. From the onset of panicle initiation to the heading time, three soil moisture treatments, WW, MD, and SD, were imposed on the plants. Plots with the WW treatment were flooded with water to a 1 to 2-cm depth by manually supplying tap water [soil water potential (Ψ_soil_) at 0 kilopascals (kPa)]. In the MD treatment, Ψ_soil_ at a 15 to 20-cm depth was maintained at − 10 to − 15 kPa (soil moisture content: 0.170–0.175 g g^− 1^), and in the SD treatment, Ψ_soil_ at a 15 to 20-cm depth was maintained at − 30 to − 35 kPa (soil moisture content: 0.145–0.150 g g^− 1^). Soil water potentials of − 10 to − 15 kPa in the MD regime and − 30 to − 35 kPa in the SD regime were chosen based on our earlier work [10, 40], which showed that a mild soil-drying regime (Ψ_soil_ = − 10 to − 15 kPa at a 15 to 20-cm depth) during the growing season did not reduce grain yield but a severe soil-drying regime (Ψ_soil_ = − 30 kPa) reduced yield by as much as 40% compared to the WW regime. Soil water potential was monitored at a 15–20-cm soil depth by five tensiometers (Institute of Soil Science, Chinese Academy of Sciences, Nanjing, China) in each plot. Tensiometer readings were recorded every 3 h from 0600 h to 1800 h. When the reading dropped to the designated value, 100 L and 60 L of tap water per plot was added manually to plots with the MD and SD treatments, respectively. Each block was protected from rain by a rain shelter consisting of a steel frame covered with a plastic sheet during rainfall events to minimize the effect of rainfall on the treatments; the shelters were removed after each rainfall event.

### Sampling

Six hundred main stems were tagged in each plot during the tillering period. One hundred young panicles were sampled from the tagged stems in each plot at the stages of SPD, PMC (the spikelet length was about 50% of the final length of a spikelet; LR: 0.4–0.3), and pollen filling (PF, the spikelet length was about 85% of the final length of a spikelet; LR: 0.2–0), respectively. In this study, the SPD, PMC and PF were chosen because the rice spikelets are differentiated at SPD, and spikelet degeneration mainly occurs at PMC or PF [5]. Young panicles were collected at each sampling time and used to measure the BRs contents, genes expression levels, and other physiological traits in young panicles.

At the heading stage, 200 young panicles that had emerged from the flag leaf sheath by two-thirds were tagged in each plot to determine the number of differentiated and degenerated spikelets per panicle. The number of differentiated spikelets was defined as the sum of the numbers of developed spikelets and degenerated spikelets. The spikelet degeneration rate was defined as the ratio of degenerated spikelets to differentiated spikelets. Fully filled grains were investigated from plants of a 1 m^2^ site (excluding the border ones) in each plot at maturity and expressed as the percentage of fully filled spikelets relative to the total number of developed spikelets (sterile spikelets, partially filled spikelets, and fully filled spikelets) in a panicle. Grain weight and grain yield were determined from plants of a 2 m^2^ site (except border ones) in each plot at maturity. The method used for these observations was described previously [10].

### Determination of leaf and panicle osmolality, photosynthetic rate, LAI, and NSC

At the onset of SPD, PMC and PF stages and when sky was clear, the osmolality of the upmost fully expanded leaves and young panicles was measured at 2-h intervals from predawn (0600 h) to evening (1800 h) in one day. The measurements were made by using a pressure osmometer (Vapro 5520, Instrumentation Consultancy Technologies, Wescor, USA), with 6 replications for each plot.

Photosynthetic rate of the upmost fully-expanded leaves was determined at PI, PMC and SPD stages using a gas exchange analyzer (Li-Cor 6400 portable photosynthesis measurement system, Li-Cor, Lincoln, NE, USA) from 0900 h to 1100 h, when the photosynthetically active radiation above the canopy was 1300–1500 μmol m^− 2^ s^− 1^. The measurements were made on the upper surface of the leaves, with six leaves in each plot. The LAI was measured at the SPD, PMC and PF stages and plants from six hills from each plot were sampled from the third row in order to minimize the border effect. At those stages, the amount of NSC in the shoots (culm + sheath + leaf blade + panicle) was determined according to the method of Yoshida et al. (1976) [41]. The rate of NSC partitioning to young panicles was calculated using the following formula:

NSC partitioning to young panicles (%) = NSC in young panicles/NSC in the shoots × 100 (1).

### Extraction and quantification of BRs

Endogenous BRs were extracted and purified using the methods of Ding et al. (2013) [42], with modifications. Briefly, each sample containing 4–6 g of fresh young panicles was ground by a tissue crusher (MM400, Retsch Corp, Haan, Germany), and 0.8 g of power was transferred into a 10-mL centrifuge tube, followed by extraction with 4 mL of acetonitrile overnight at 20 °C. Extraction, dehydration and double-layered solid phase extraction (DL/SPE) were performed according to the method of Chen et al. (2009) [43]. The quantification of BRs was performed using high-performance liquid chromatography-electrospray ionization-tandem mass spectrometry (HPLC-ESI-MS/MS) according to the protocol of Ding et al. (2013) [42]. The BRs were quantified using a calibration curve with known amounts of standards and based on the ratios of the summed area of the multiple reaction monitoring (MRM) transitions for BRs. Data acquisition and analysis were performed using the Xcalibur Data System (Thermo Fisher Scientific, Waltham, MA, USA). The BRs level was expressed as pmol g^− 1^ dry weight (DW). In this study, 24-epicastasterone (24-epiCS) and 28-homobrassinolide (28-homoBL) were analyzed because they are the most important BRs in rice plants due to their high biological activity [44].

### Determination of genes expression levels

The expression levels of key BRs synthesis gene *OsD11* [45], AsA synthesis and metabolic related genes *OsMPG1* (encodes GMPase), *OsDHAR1*, *OsMDHAR3*, *OsAPX1* and *OsAPX2* [46–49], a key sugar metabolism gene *OsCSA* [33] in rice young panicles were analyzed at SPD, PMC and PF stages. The expression levels of key inflorescence development genes *OsAPO2* and *OsTAW1* in young panicles were determined at the SPD stage since they are mainly expressed at transition from inflorescence meristem to spikelet meristem in rice [7]. Transcript levels of the genes were measured by qRT-PCR using an iCycler (Bio-Rad, Hercules, CA, USA) with iQ SYBR Green Supermix (Bio-Rad). The gene accession numbers and gene-specific primer pairs used for qRT-PCR are listed in Additional file 1: Table S1. Rice *ACTIN1* was used as an internal reference for all analyses. Three replicates were performed for each sample.

### Determination of contents of AsA and H_2_O_2_, and activities of APX, DHAR and MDHAR

Measurement of AsA content was performed by a HPLC system (Waters 2695 separation module, Waters Corp, Millford, MA, USA) according to the method of Wang et al. (2013) [22]. The AsA content was calculated by comparison with the values obtained from a standard curve. The H_2_O_2_ content in young panicles was measured using the method of Rao et al. (2000) [50]. Both AsA and H_2_O_2_ contents were expressed as μmol g^− 1^ DW.

The activities of APX, DHAR and MDHAR were measured using the methods of Gomez and Lajolo (2010) [51] and Hou and Lin (1997) [52], respectively. Oxidation or reduction of 1 μmol AsA per gram dry weight (DW) of samples per min at 25 °C was defined as a unit of the activity for APX or for DHAR, and oxidation of 1 μmol nicotinamide adenine dinucleotide (NADH) per gram dry weight of samples per min at 25 °C as a unit of the activity for MDHAR. Activities of the three enzymes were expressed as unit g^− 1^ DW min^− 1^.

### Construction of transgenic line (*D11-RNAi*, called *DR-1*) and soil moisture treatments

The methods of constructing the artificial RNAi vector and transformation were as described by Zhu et al. (2015) [33]. Seeds of the WT and *DR-1* were sown in the field, and thirty-day-old seedlings were then transplanted into porcelain pots (25-cm height, 20-cm diameter, and 7.75-L volume for each pot) with one seedling per pot during the rice growing season. Each pot was filled with 7.0 kg of sandy loam soil (Typic Fluvaquent, Entisol, US classification), and the soil composition was as described above. Both the WW (Ψ_soil_ = 0 kPa) and MD (Ψ_soil_ = − 10 to − 15 kPa) treatments were imposed from the onset of panicle initiation to the heading time. The treatment details, water management, and rain prevention were the same as those in the field experiment. Each treatment consisted of 80 pots and 100–120 young panicles were sampled at the SPD stage, 20–30 young panicles were sampled at PMC and PF stages to measure the contents of BRs (24-epiCS + 28-homoBL), AsA and H_2_O_2_, and relative expression levels of *OsD11*, *OsCSA*, *OsMPG1*, *OsDHAR1*, *OsMDHAR3*, *OsAPX1* and *OsAPX2*, and activities of APX, DHAR and MDHAR in young panicles. The expression levels of *OsAPO2* and *OsTAW1* in young panicles were determined at the SPD stage. At the three stages, the amount of NSC in the shoots (culm + sheath + leaf blade + panicle) was determined. The number of differentiated or degenerated spikelets in a panicle was determined from 60 panicles in each treatment at the heading stage. The methods used for the determinations were the same as those described above.

### Chemical application

At the onset of SPD, 10 nmol L^− 1^ BRs (24-epiCS + 28-homoBL) (eBRs), 2 mmol L^− 1^ AsA (eAsA), or 2 mmol L^− 1^ AsA + 10 mmol L^− 1^ sucrose [e (AsA + sucrose)] was applied to the panicles of *DR-1* or SD treated-YD-6, and 10 nmol L^− 1^ BRZ (eBRZ) or 50 mmol L^− 1^ H_2_O_2_ (eH_2_O_2_) was applied to the panicles of WT or YD-6 in the WW treatment by carefully injecting the solution ~ 2 cm from the basal stem with a 1-mL syringe (Ultra-Fine Needle Insulin Syringe, Becton, Dickinson and Company). The injection site is shown in the diagram (Fig. 7a). Physical damage caused by the injection event was not observed since the pinhole was very fine (0.33 mm). The chemicals were applied daily for 3 days at the onset of the SPD stage with 0.5 mL of solution per panicle per day. All the solutions contained ethanol at a final concentration of 0.05% (v/v). Control plants (CK) received the same volume of deionized water containing the same concentration of ethanol. The *DR-1* and WT plants were pot-grown as described above, and the WW treatment was imposed from the onset of panicle initiation to the heading time, with 40 replicate pots. The cultivar YD-6 was field grown as described above under the WW and SD conditions with three replications. Each chemical treatment included 80 young panicles as replicates, and these panicles were tagged.

In each chemical treatment, the expression levels of *OsAPO2* and *OsTAW1* in young panicles were determined at the SPD stage, contents of AsA, H_2_O_2_ and NSC, activities of APX, DHAR and MDHAR, expression levels of *OsCSA*, *OsMPG1*, *OsDHAR1*, *OsMDHAR3*, *OsAPX1* and *OsAPX2* in young panicles were determined at the PMC stage, and contents of BRs (24-epiCS + 28-homoBL) were determined at both SPD and PMC stage. The number of differentiated or degenerated spikelets in a panicle was determined at the heading stage. The methods used for the determinations were the same as those described above.

### Statistical analysis

Analysis of variance was performed using the SAS/STAT statistical analysis package (version 9.2, SAS Institute, Cary, NC, USA). Data from each sampling date were analyzed separately. Means were tested by a least significant difference (LSD) test at the *P* = 0.05 level (LSD_0.05_).

## Supplementary information

**Additional file 1: Table S1.** List of the primers used for qRT-PCR analyses. **Fig. S1** Changes in relative expression levels of genes in young panicles of the rice cultivar YD-6 under well-watered, moderate soil-drying and severe soil-drying treatments. **Fig. S2** Changes in relative expression levels of genes in young panicles of WT and *DR-1* under well-watered and moderate soil-drying treatments. **Fig. S3** Changes in relative expression levels of key rice inflorescence development genes in young panicles of YD-6, ZH10 (WT) and *OsD11* RNAi line (*DR-1*) under various soil moisture treatments. **Fig. S4** Effect of exogenous brassinosteroids and brassinazole on relative expression levels of ascorbic acid synthesis and cycle, or sugar metabolism genes in young panicles of YD-6, ZH10 (WT) and *OsD11* RNAi line (*DR-1*). **Fig. S5** Effect of exogenous brassinosteroids and brassinazole on relative expression levels of key rice inflorescence development genes in young panicles of YD-6, ZH10 (WT) and *OsD11* RNAi line (*DR-1*). **Fig. S6** Effect of exogenous brassinosteroids and brassinazole on physiological traits and spikelet development of rice cultivar YD-6 under well-watered and severe soil-drying treatments. **Fig. S7** Effect of exogenous H_2_O_2_, ascorbic acid and sucrose on endogenous H_2_O_2_ content in young panicles, spikelet differentiation and degeneration of YD-6, ZH10 (WT) and *OsD11* RNAi line (*DR-1*).

## Data Availability

All relevant data are within this article and its Additional files.

## Abbreviations

24-epiCS: 24-epicastasterone
28-homoBL: 28-homobrassinolide
AO: Ascorbic acid oxidase
AOS: Antioxidant system
APX: Ascorbic acid peroxidase
AsA: Ascorbic acid
BRS: Brassinosteroids
BRZ: Brassinazole
DHAR: Dehydroascorbate reductase
GMPase: GDP-D-mannose pyrophosphorylase
GSH: Glutathione
GSSH: oxidized Glutathione
LAI: Leaf area index
MD: Moderate soil-drying
MDHAR: Monodehydroascorbate reductase
NSC: Nonstructural carbohydrates
PF: Pollen filling
PMC: Pollen mother cell meiosis
ROS: Reactive oxygen species
SD: Severe soil-drying
SPD: Spikelet primordium differentiation
WW: Well-watered
