# Supplementary material for: Role of brassinosteroids in rice spikelet differentiation and degeneration under soil-drying during panicle development
Source: BMC Plant Biol. 2019 Sep 18;19:409. doi: 10.1186/s12870-019-2025-2 (PMC6749693; doi:10.1186/s12870-019-2025-2)
Supplement: Supplementary file 1 — Additional file 1: Table S1. List of the primers used for qRT-PCR analyses. Fig. S1 Changes in relative expression levels of genes in young panicles of the rice cultivar YD-6 under well-watered, moderate soil-drying and severe soil-drying treatments. Fig. S2 Changes in relative expression levels of genes in young panicles of WT and DR-1 under well-watered and moderate soil-drying treatments. Fig. S3 Changes in relative expression levels of key rice inflorescence development genes in young panicles of YD-6, ZH10 (WT) and OsD11 RNAi line (DR-1) under various soil moisture treatments. Fig. S4 Effect of exogenous brassinosteroids and brassinazole on relative expression levels of ascorbic acid synthesis and cycle, or sugar metabolism genes in young panicles of YD-6, ZH10 (WT) and OsD11 RNAi line (DR-1). Fig. S5 Effect of exogenous brassinosteroids and brassinazole on relative expression levels of key rice inflorescence development genes in young panicles of YD-6, ZH10 (WT) and OsD11 RNAi line (DR-1). Fig. S6 Effect of exogenous brassinosteroids and brassinazole on physiological traits and spikelet development of rice cultivar YD-6 under well-watered and severe soil-drying treatments. Fig. S7 Effect of exogenous H2O2, ascorbic acid and sucrose on endogenous H2O2 content in young panicles, spikelet differentiation and degeneration of YD-6, ZH10 (WT) and OsD11 RNAi line (DR-1). [file 12870_2019_2025_MOESM1_ESM.doc]

**Supplementary information**

**Role of brassinosteroids in rice spikelet differentiation and degeneration under soil-drying during panicle development**

Weiyang Zhang ^1, 2^, Jiayan Sheng ^1, 2^, Yunji Xu ^3^, Fei Xiong ^4^, Yunfei Wu ^4^, Weilu Wang^1, 2^, Zhiqin Wang ^1, 2^, Jianchang Yang ^1, 2, 3*^ and Jianhua Zhang ^5^

^1^ *Jiangsu Key Laboratory of Crop Genetics and Physiology/ Jiangsu Key Laboratory of Crop Cultivation and Physiology, Agricultural College of Yangzhou University, Yangzhou 225009, China*

*^2^ Jiangsu Co-Innovation Center for Modern Production Technology of Grain Crops, Yangzhou University, Yangzhou 225009, China* *Joint International Research*

^3^ *Laboratory of Agriculture and Agri-Product Safety, Yangzhou University, Yangzhou, Jiangsu, 225009, China*

^4^ *College of Bioscience and Biotechnology, Yangzhou University, Yangzhou, Jiangsu, 225009, China*

^5^ *Department of Biology, Hong Kong Baptist University, Hong Kong, China*

* To whom correspondence should be addressed:

**Jianchang Yang**, Tel: +86 514 87979317, Fax: +86 514 87324276. E-mail: [jcyang@yzu.edu.cn](mailto:jcyang@yzu.edu.cn)

**Table S1 List of the primers used for q**RT-PCR analyses.

| **Gene name** | **Accession number** | **Primer pairs** |
| --- | --- | --- |
| ***DWARF 11* (*OsD11*)** | AB158759 | F: 5’-TTGGGTCATGGCATGGCAAGAGCAAGGA-3’  R: 5’-TTGTTGCTGGAGCCAGCATTCCTCCTCT-3’ |
| ***R2R3-TYPE MYB GENE* (*OsCSA*)** | [AK107461](http://www.ncbi.nlm.nih.gov/nuccore/AK107461) | F: 5’TGTGCGTTTGGAAATCAACA3’  R: 5’GCGTCATGGACAATGAACAC3’ |
| ***MANNOSE-1-PHOSPHATE GUANYL TRANSFERASE* *1* (*OsMPG1*)** | AK061976 | F: 5’-GTCATGTGAACTAACCCTCC-3’  R: 5’-GAGTTTCTTCTGGTCCTCTTG-3’ |
| ***CYTOSOLIC DEHYDROASCORBATE REDUCTASE 1* (*OsDHAR1*)** | [AY074786](http://www.ncbi.nlm.nih.gov/nuccore/AY074786) | F: 5’-AGGTGCCCTACGAGATGAAGC -3’  R: 5’-AATCCATTTGCCATCACCACC -3’ |
| ***ASCORBATE PEROXIDASE GENE 1* (*OsAPX1*)** | [AK061841](http://www.ncbi.nlm.nih.gov/nuccore/AK061841) | F: 5’-TGTCCTTGTCACTCAAACCCATC-3’  R: 5’-GACCAACTTCCCATCCTCTCCTA-3’ |
| ***ASCORBATE PEROXIDASE GENE 2* (*OsAPX2*)** | [AB053297](http://www.ncbi.nlm.nih.gov/nuccore/AB053297) | F: 5’-CCGACGGTGAGCGATGAGT -3’  R: 5’-CGAAGGTGCCAGCAGAGTG -3’ |
| ***MONODEHYDROASCORBATE REDUCTASE 3* (*OsMDHAR 3*)** | D85764 | F: 5’-TTGTTGGTGTTGGTGTTGGG-3’  R: 5’-GCAGGCTGTAAAGGCAATCA-3’ |
| ***ABERRANT PANICLE ORGANIZATION 2 (OsAPO2)*** | AB005620 | F: 5’-AGGTGCAATCCATGGCTAAG-3’  R: 5’-CGCGTAGCAGTGCACGTAGT-3’ |
| ***TAWAWA1 (OsTAW1)*** | AB512494 | F: 5’-CTAGTTACTCCACTCCACTC-3’  R: 5’-GTAGTTTTGCTAGTAGCAAG-3’ |
| ***ACTIN1*** | X16280 | F: 5'-CCTTCAACACCCCTGCTATG-3'  R: 5'-CAATGCCAGGGAACATAGTG-3' |

Note: F = forward primer; R = reverse primer.


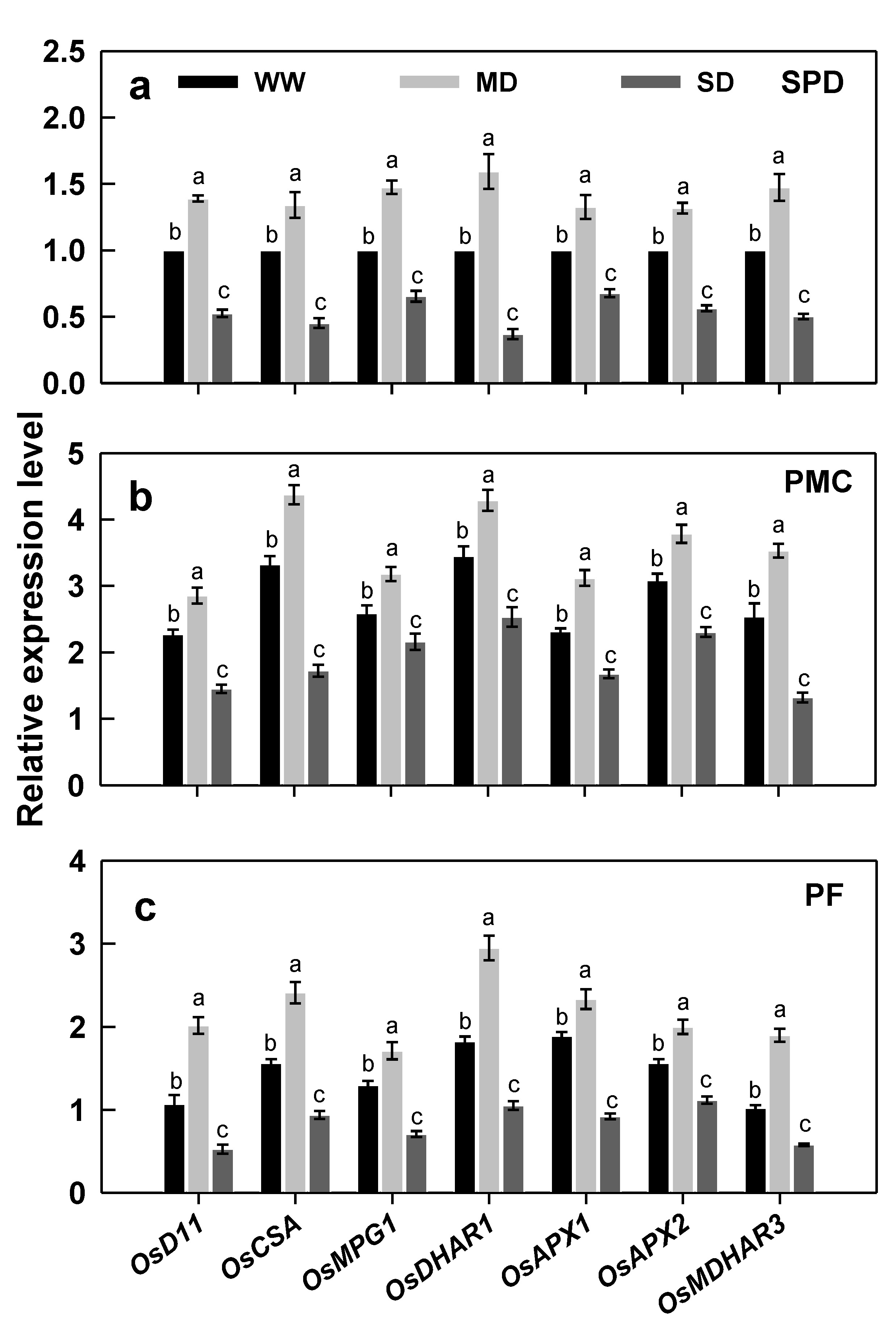


**Figure S1 Changes in relative expression levels of genes (a-c) in young panicles of the rice cultivar YD-6 under well-watered (WW), moderate soil-drying (MD) and severe soil-drying (SD) treatments.**

SPD, PMC, and PF represent spikelet primordium differentiation, pollen mother cells meiosis, and pollen filling, respectively. Vertical bars represent ± standard error of the mean (n = 6) where these exceed the size of the symbol. Different letters above the bars indicate the least significant difference at *P* = 0.05 within the same gene.


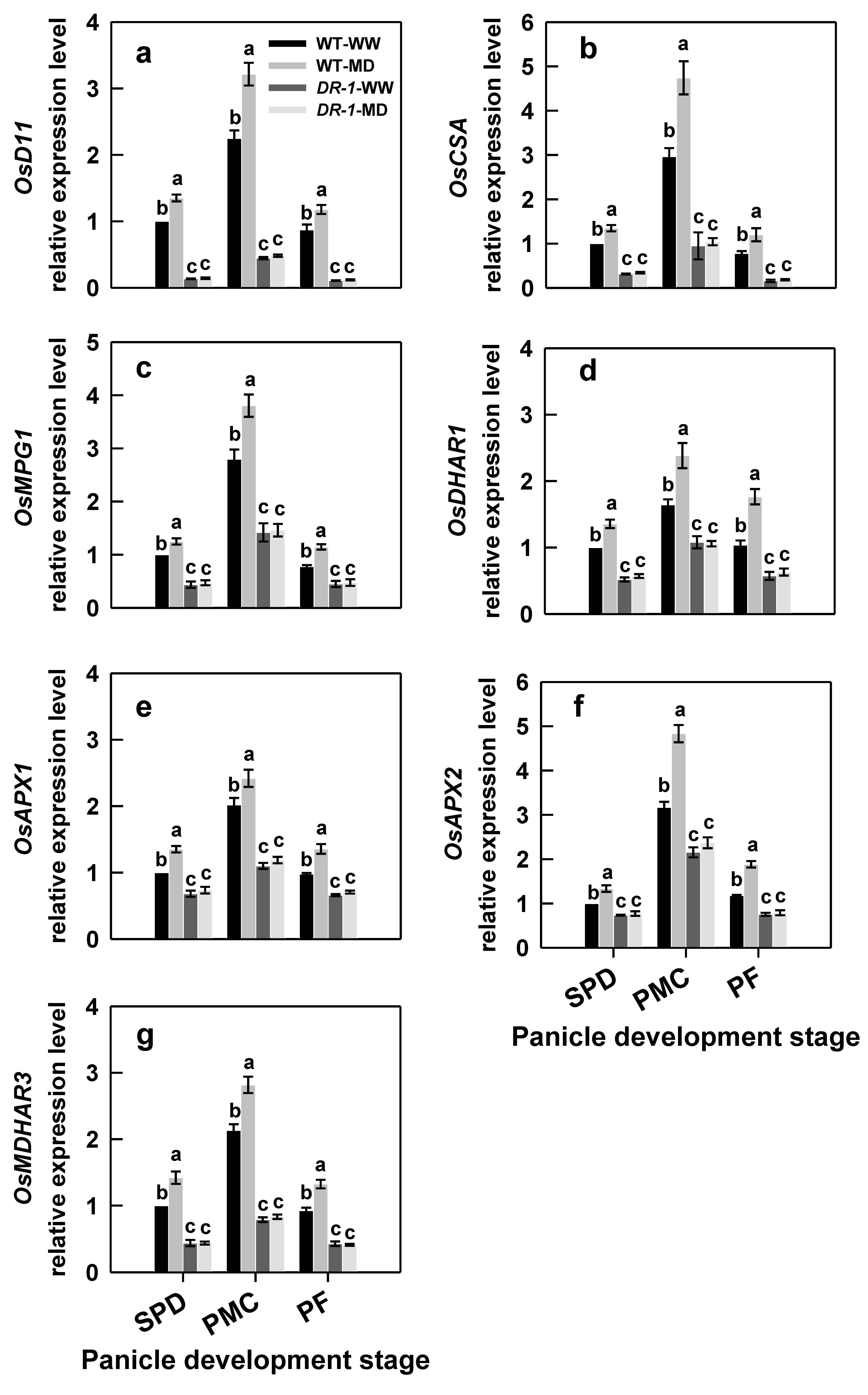


**Figure S2 Changes in relative expression levels of genes in young panicles of WT and *DR-1* under well-watered (WW) and moderate soil-drying (MD) treatments.**

SPD, PMC, and PF represent spikelet primordium differentiation, pollen mother cells meiosis, and pollen filling, respectively. Vertical bars represent ± standard error of the mean (n = 6) where these exceed the size of the symbol. Different letters above the bars indicate the least significant difference at *P* = 0.05 within the measurement date.


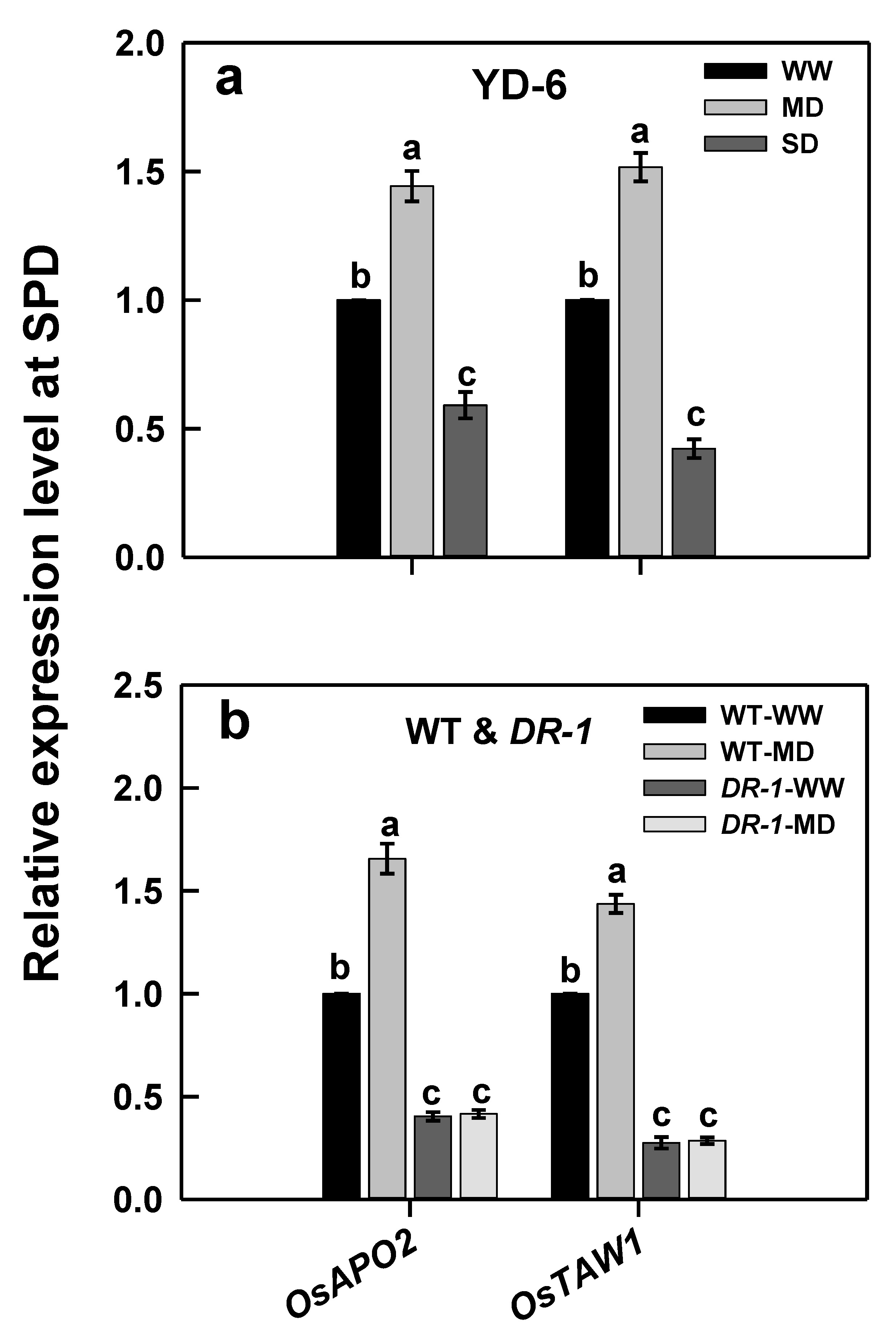


**Figure S3 Changes in relative expression levels of key rice inflorescence development genes in young panicles of YD-6 (a), ZH10 (WT) and *OsD11* RNAi line (*DR-1*) (b) under various soil moisture treatments.**

SPD represent spikelet primordium differentiation. Vertical bars represent ± standard error of the mean (n = 6) where these exceed the size of the symbol. Different letters above the bars indicate the least significant difference at *P* = 0.05 within the same gene.


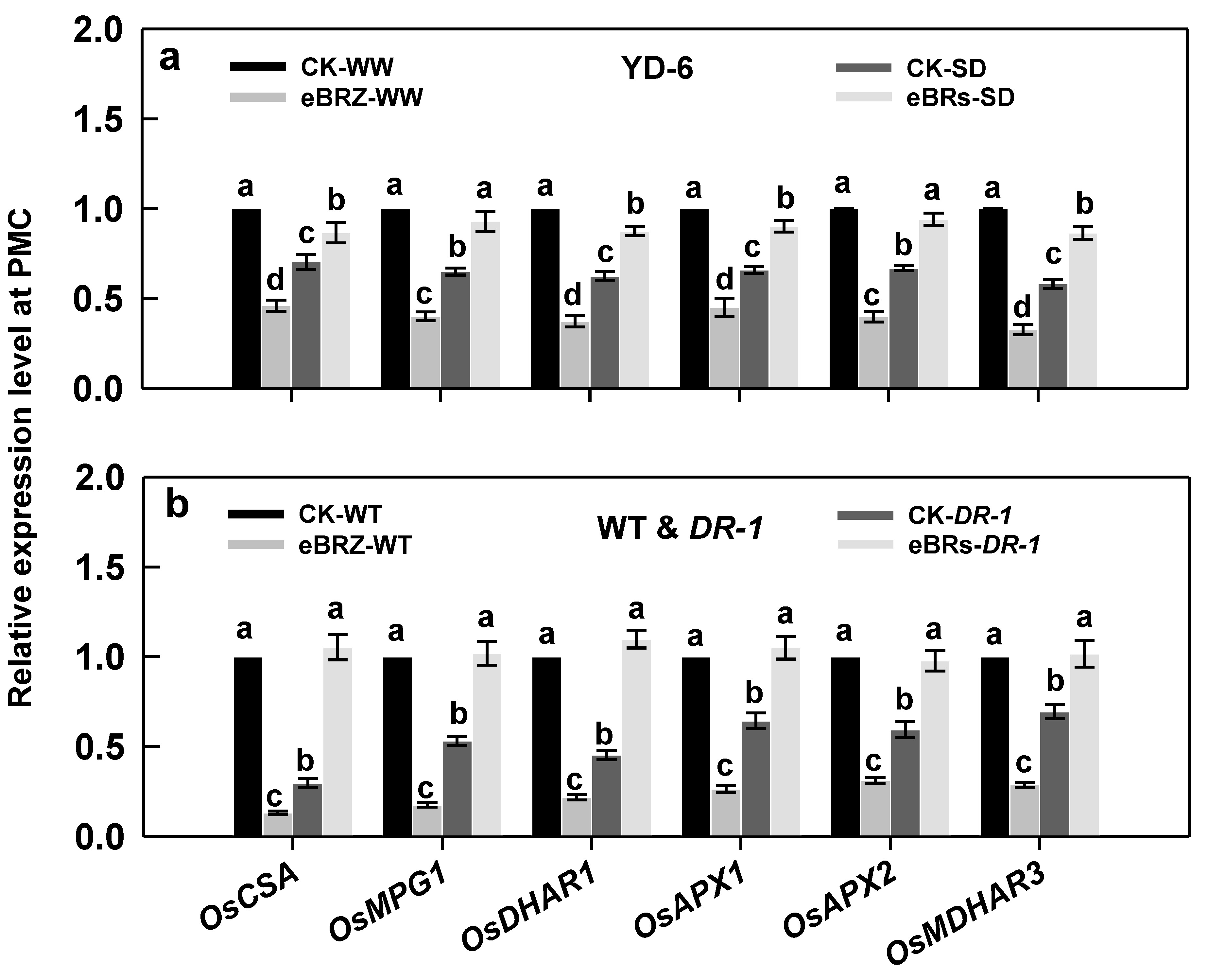


**Figure S4 Effect of** [**exogenous**](javascript:;) **brassinosteroids (BRs) and brassinazole (BRZ) on relative expression levels of ascorbic acid (AsA) synthesis and cycle, or sugar metabolism genes in young panicles of YD-6 (a), ZH10 (WT) and *OsD11* RNAi line (*DR-1*) (b).**

PMC represent pollen mother cells meiosis. **CK**: Panicles received deionized water; **eBRZ**: Panicles received 10 nmol L^-1^ brassinazole (BRZ, an inhibitor of BRs biosynthesis); **eBRs**: Panicles received 10 nmol L^-1^ BRs (24-epiCS + 28-homoBL). Vertical bars represent ± standard error of the mean (n = 6) where these exceed the size of the symbol. Different letters above the bars indicate the least significant difference at *P* = 0.05 within the same gene.


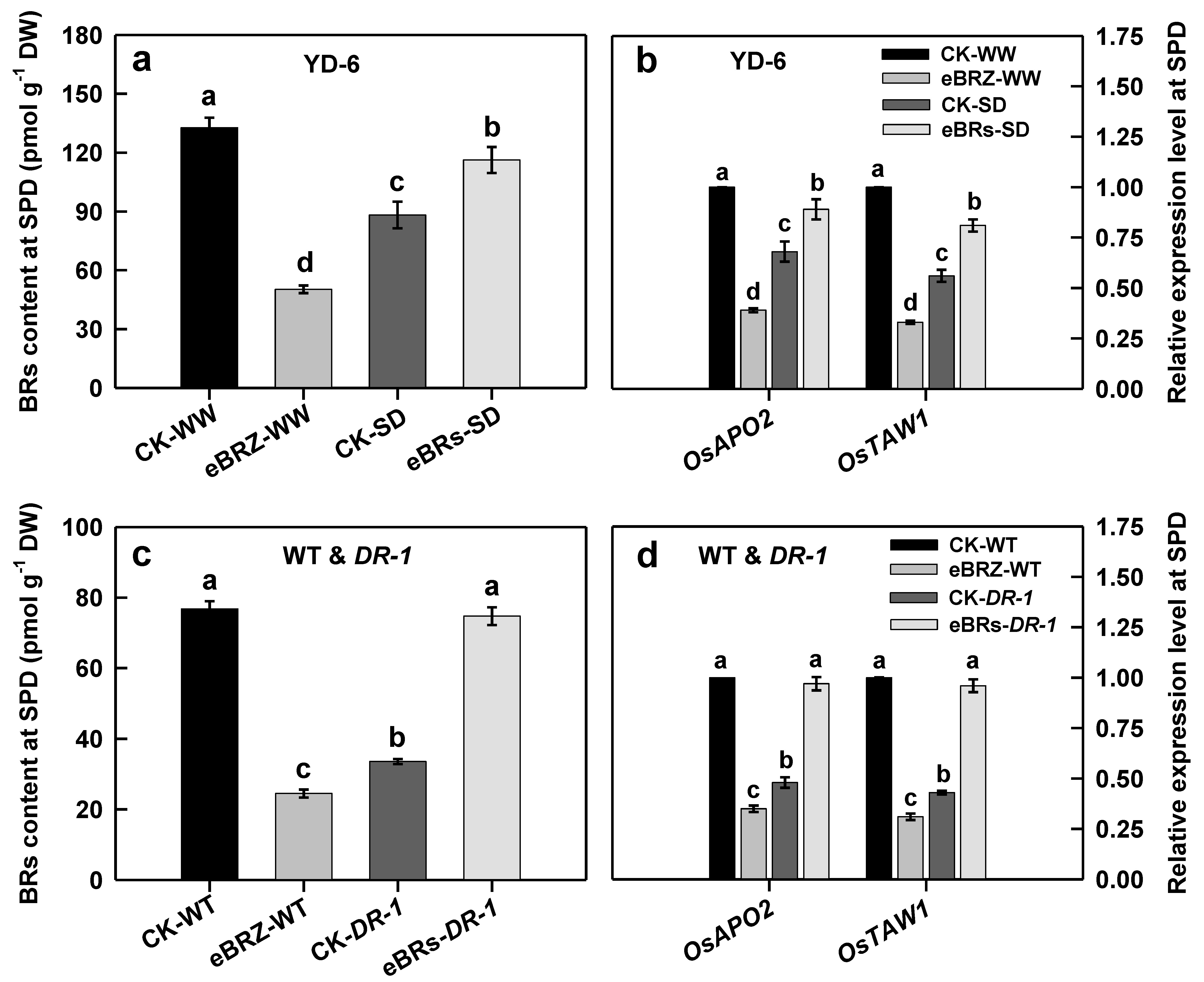


**Figure S5 Effect of** [**exogenous**](javascript:;) **brassinosteroids (BRs) (24-epiCS + 28-homoBL) and brassinazole (BRZ) on relative expression levels of key rice inflorescence development genes in young panicles of YD-6 (a, b), ZH10 (WT) and *OsD11* RNAi line (*DR-1*) (c, d).**

SPD represent spikelet primordium differentiation. **CK**: Panicles received deionized water; **eBRZ**: Panicles received 10 nmol L^-1^ brassinazole (BRZ, an inhibitor of BRs biosynthesis); **eBRs**: Panicles received 10 nmol L^-1^ BRs (24-epiCS + 28-homoBL). Vertical bars represent ± standard error of the mean (n = 6) where these exceed the size of the symbol. Different letters above the bars indicate the least significant difference at *P* = 0.05 within the same item (a, c) or gene (b, d).


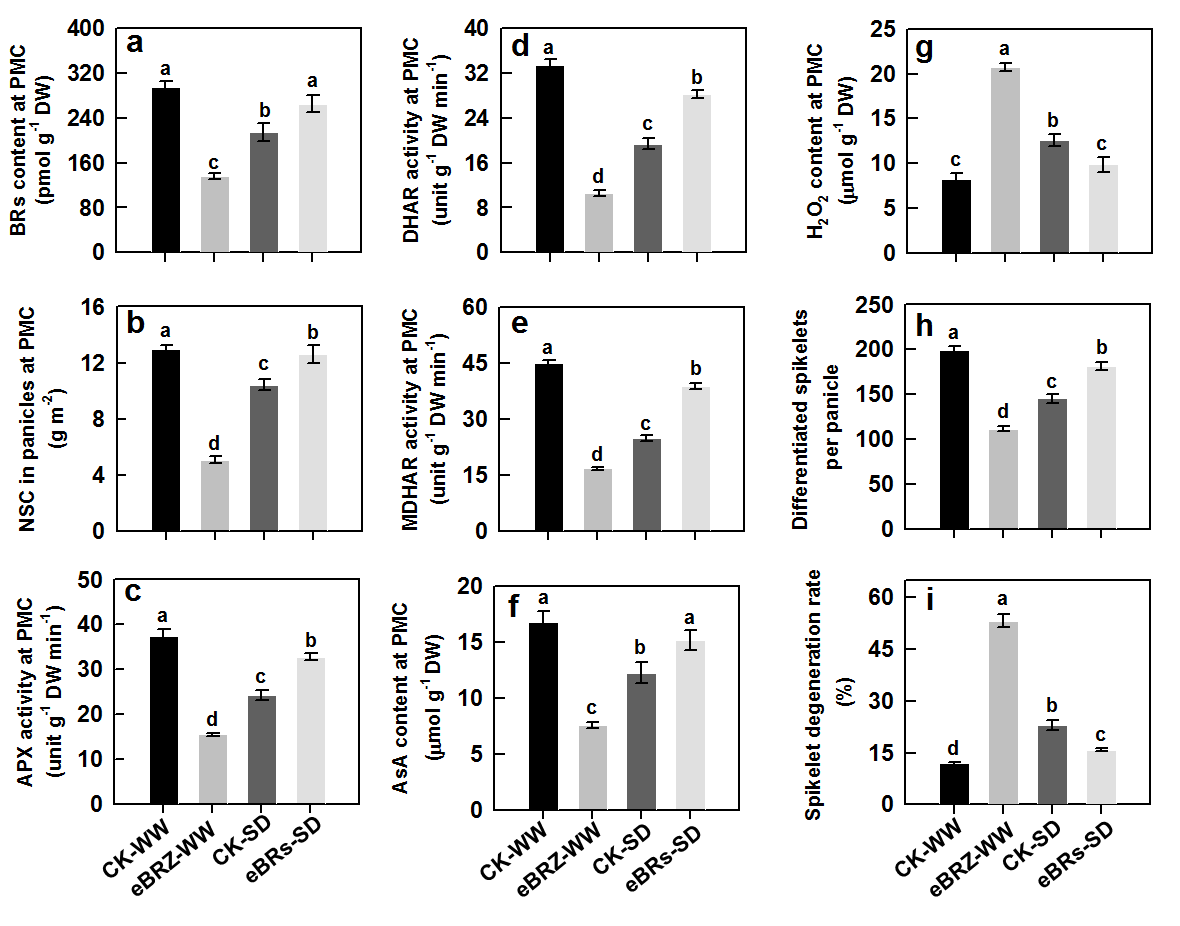
**Figure S6 Effect of exogenous brassinosteroids (BRs) (24-epiCS + 28-homoBL) and brassinazole (BRZ) on physiological traits and spikelet development of rice cultivar YD-6 under various soil moisture treatments.**

PMC represent pollen mother cells meiosis. **CK**: Panicles received deionized water; **eBRZ**: Panicles received 10 nmol L^-1^ brassinazole (BRZ, an inhibitor of BRs biosynthesis); **eBRs**: Panicles received 10 nmol L^-1^ BRs (24-epiCS + 28-homoBL). Vertical bars represent ± standard error of the mean (n = 6) where these exceed the size of the symbol. Different letters above the bars indicate the least significant difference at *P* = 0.05 within the same item.


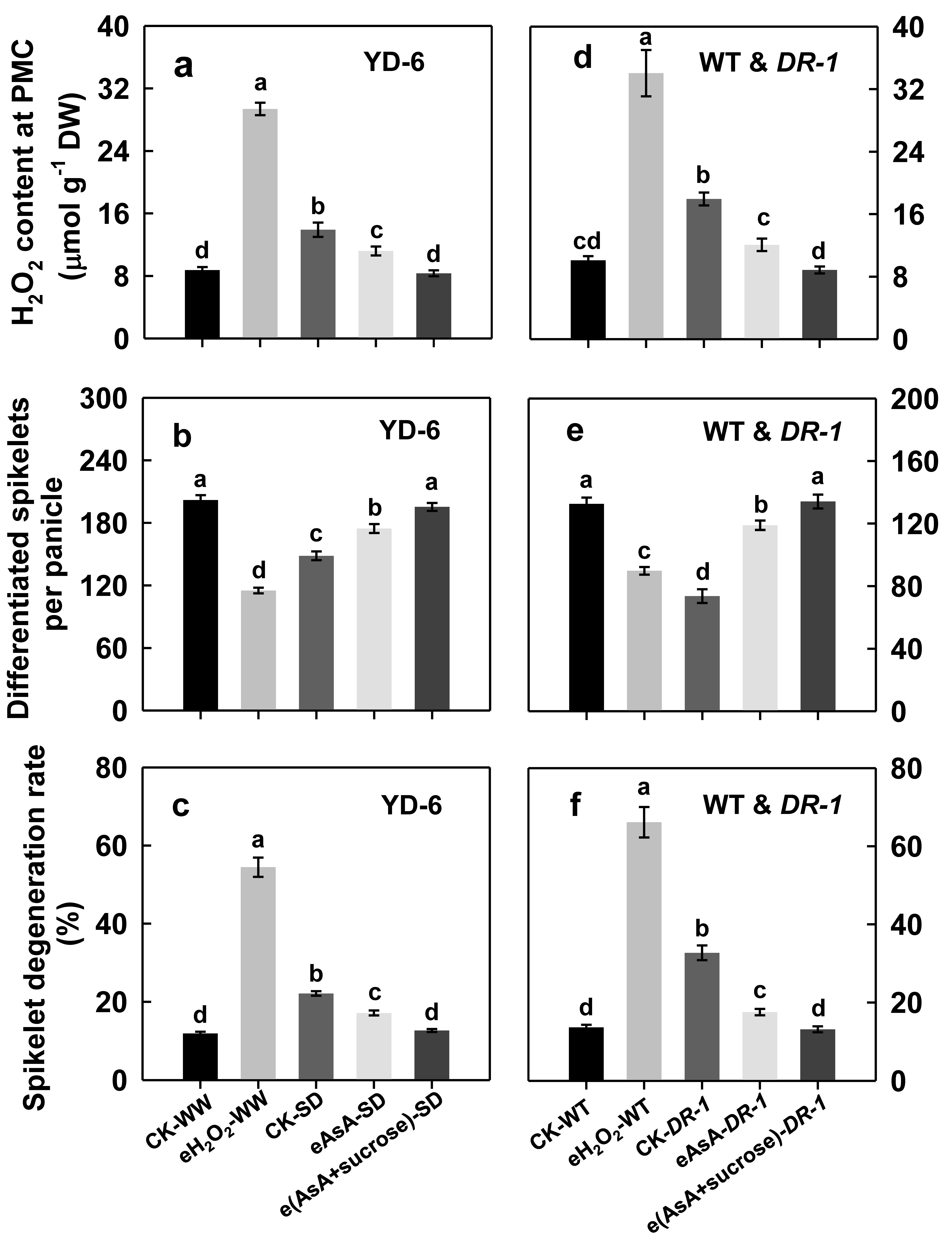


**Figure S7 Effect of exogenous H_2_O_2_, ascorbic acid (AsA) and** **sucrose on** **endogenous H_2_O_2_ content in young panicles, spikelet differentiation and degeneration of YD-6 (a-c), ZH10 (WT) and *OsD11* RNAi line (*DR-1*) (d-f).**

**CK**: Panicles received deionized water; **eH_2_O_2_**: Panicles received 50 mmol L^-1^ H_2_O_2_; **eAsA**: Panicles received 2 mmol L^-1^ AsA; **e(AsA+sucrose)**: 2 mmol L^-1^ AsA + 10 mmol L^-1^ sucrose. Vertical bars represent ± standard error of the mean (n = 6) where these exceed the size of the symbol. Different letters above the bars indicate the least significant difference at *P* = 0.05.
